# A Comparison of Infection Venues of COVID-19 Case Clusters in Northeast China

**DOI:** 10.3390/ijerph17113955

**Published:** 2020-06-03

**Authors:** Pengcheng Zhao, Nan Zhang, Yuguo Li

**Affiliations:** 1 Department of Mechanical Engineering, The University of Hong Kong, Hong Kong 999077, China; zhangnan@hku.hk (N.Z.); liyg@hku.hk (Y.L.); 2 School of Public Health, The University of Hong Kong, Hong Kong 999077, China

**Keywords:** case report, cluster, infection location, COVID-19, Northeast China

## Abstract

The world has been suffering from the COVID-19 pandemic since late 2019. In this study, we compared various types of infection locations in which COVID-19 cases clustered, based on the data from three adjacent provinces in Northeast China. The collected data include all officially reported cases in this area until 8 March 2020. We explored the associations between the cases and the frequency of infection locations. The COVID-19 epidemic situation was worse in Heilongjiang Province than in Liaoning and Jilin Provinces. Most clustered cases occurred in individual families and/or between relatives. The transmission in public venues served as a hub for transmitting the disease to other families and results in new clusters. The public transport spread the infection over long distances by transporting infected individuals, and most infections did not seem to occur within vehicles. This field study shows the effect of indoor environments on SARS-CoV-2 transmission and our data may be useful in developing guidance for future disease prevention and control.

## 1. Introduction

A novel coronavirus pneumonia (COVID-19) emerged in Wuhan, Hubei Province, China in late 2019, and has swept around the world [1,2,3]. Its transmissibility between people has been reported to exceed that of SARS-CoV-1 [4,5,6] and MERS-CoV [7,8]. Several studies have investigated SARS-CoV-2 transmission by the statistical analysis of outbreaks on large scales, such as entire countries [3,9,10,11] or by case studies of a family [12,13,14,15,16,17] or a specific group of people, such as the elderly [18] and healthcare workers [19]. However, few studies have focused on the characteristics of SARS-CoV-2 transmission for a regional epidemic situation in a medium-scale area, with an analysis accounting for local policy and government actions. Thus, we propose choosing a region with a sufficient number of COVID-19 cases and their detailed case reports to illustrate the role of different types of location in person-to-person transmission of this disease. In the COVID-19 outbreak, the Chinese health authorities made their case investigation data publicly available [8,20]. These data present a good opportunity to investigate the roles of various infection locations in SARS-CoV-2 transmission.

Previous viral outbreaks have reportedly involved transmission in various types of location, such as SARS-CoV-1 in a residential estate [21], MERS-CoV in a hospital [7], norovirus on mass transport [22] and influenza virus A in a market [23], with various transmission routes, including the fomite [24], large droplet [25] and airborne [26] routes. SARS-CoV-2, as a novel coronavirus, has also been reported to be transmitted in various locations, such as a shopping mall [27], a cruise ship [28], healthcare settings [19] and homes [15,16], with various possible transmission routes [29]. However, direct determination of the exact infection location is difficult in most situations when there is no complete and detailed record of a newly infected patient’s itinerary before the case was confirmed [30]. Only when we know the details of a patient’s movement can we effectively determine the infection source [31]. For some cases, the infection source cannot be identified during initial tracking of the case’s activities, unless trajectories of all individuals are tracked in public places, such as buses, restaurants and shops.

In this study, we analysed the reported cases in Liaoning, Jilin and Heilongjiang, which are three adjacent provinces in Northeast China. The travel records of all confirmed patients in these three provinces have been posted online by local health authorities via official websites, social media or newspapers. For details, please refer to Table S1. This provides an opportunity to analyse the reported cases within a city or over provinces. The population inflow from southern China before the 2020 Lunar New Year (25 January) is known to have mainly come to these three provinces [32], which helps in identifying the imported cases into the studied area.

We first summarised all 712 COVID-19 cases in the three provinces before 8 March 2020, and identified the relationships among the reported cases based on case reports. We also clustered the cases according to meeting locations of the involved individuals. Based on the collected and deduced information, we compared the epidemic situations between cities and/or provinces and enumerated the cases for the various location clusters.

## 2. Methods

### 2.1. Data Collection and Preparation

We collected personal information (surname, age, sex, infection source) and itinerary reports of all cases confirmed before 8 March 2020 in the three provinces of Northeast China. These cases were reported daily by the local health authorities of 37 cities via their websites, with a few cases published via social media or online newspapers, as listed in Table S1. The cases in each city (large city or prefecture regionalised by the State Council [33]; the administrative region containing counties, cities, municipal districts and townships) were sequentially numbered according to the timing of notification (or the original order in the city’s report, usually according to the date of confirmation). A total of 712 cases were collected in the three provinces (Liaoning, 126 cases; Jilin, 92 cases; Heilongjiang, 494 cases). We determined any related cases, including family members, relatives, friends and strangers in close contact [34]. Note that the total number of cases in this study was based on the governments’ daily reports. In some cities, we observed that the final reported number of confirmed cases was less than the sum of the daily reported cases. This occurred as some of the cases were later identified to belong to other regions outside the three provinces. In other situations, a few asymptomatic cases had been originally counted in the daily report, but were finally excluded from the statistics.

When determining the potential relationships among cases, we noted that some cases had the same address and detailed itinerary, but their potential relationships were not directly noted in their case reports. We identified those cases as possible clusters in families. For any case pairs with a relationship (directly collected from case reports or identified by searching the report details) to the existing cases, the relationship is specified in Table S1.

We also listed the locations where each case stayed and was likely to meet an infection source (in Table S1). We summarised the frequently visited locations and categorised them according to the involved activities between people from an estimated high infection probability to an estimated low infection probability, as shown in Table 1, by assuming that dining activities and long-term or repeated close contact between people can increase the probability of the disease transmission [22,34].

### 2.2. Identification of Case Clusters

**Figure 1:**
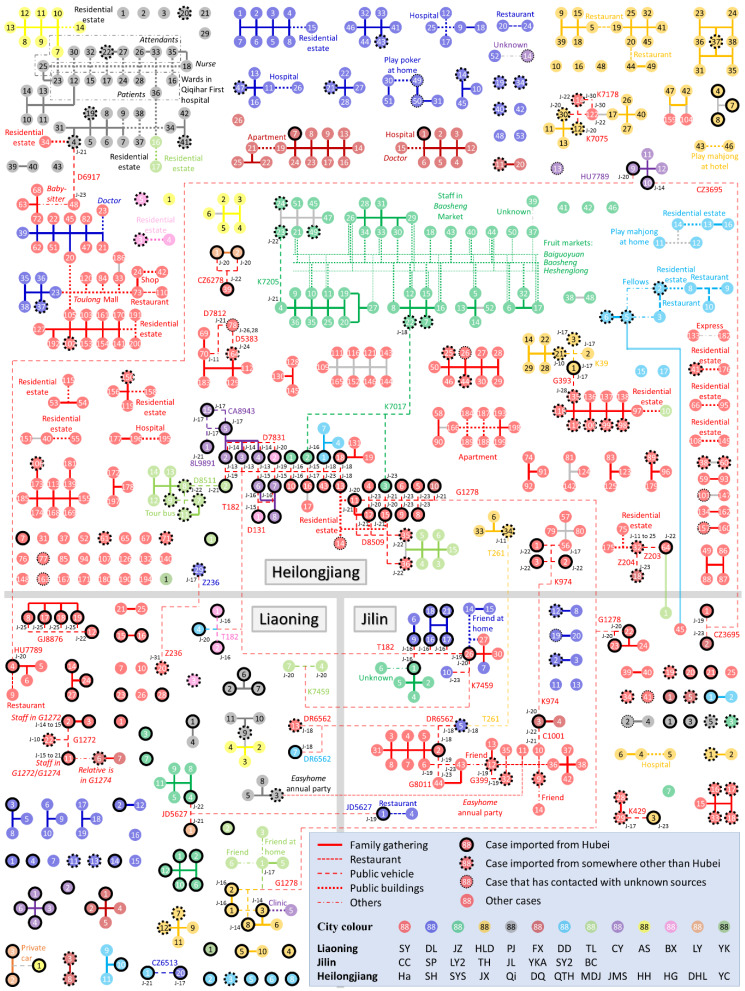
Summary of all COVID-19 cases before 8 March 2020 in Liaoning, Jilin and Heilongjiang Provinces. The cases in different cities in each province are shown by colour and are clustered according to their clustering location. Cases connected with light grey lines are possible clusters in families, based on their same itinerary and address. The cities’ names corresponding to the abbreviations are listed in Table S1. The locations for the clusters outside the home and the flight and train numbers along with the cases’ travelling dates are specified on the diagram.

We summarise each of the cases and identify their relationships in Figure 1, based on the information summarised in Table S1. Icons of the 712 cases are located in the three provinces in Figure 1, differentiated by colour according to their reported cities with their serial numbers in the cities. The cases were categorized into four groups: (I) imported from Hubei Province (black solid edge); (II) imported from anywhere outside the three provinces other than Hubei, including other countries (dashed edge); (III) locally infected cases who had contact with an unknown COVID-19 source (thin-dot edge); and (IV) locally infected cases with no report of contact with any COVID-19 sources (no edge). In addition, the cases were clustered and connected by five types of line corresponding to the five categories of clustering location in Table 1. Figure 1 specifies all locations outside the home where case clustering occurred, and all train or flight numbers are marked along with the cases’ travel date. Note that clusters solely within families in public locations (i.e., the locations of the last four categories in Table 1) are not marked on Figure 1, because the potential infection within families was more likely to occur at home, so family itineraries outside the home may be assumed to play an insignificant role in investigating the location of infection clusters. Further statistical operations were performed according to the information in Figure 1.

### 2.3. Epidemic Situation in Different Regions

To further understand the characteristics of the cases in the three provinces, a series of parameters were defined and counted as follows.

First, the daily increase in the number of cases was searched for each province during the period between 18 January 2020 and 8 March 2020. These statistical data were obtained from each provincial Health Commission (websites are listed in Table S1), as not all collected case reports had the confirmation date.

Second, to compare epidemic prevention and control between administrative regions, the case density (number of cases per million people) and the imported ratio (ratio of the number of imported cases to the total number of cases) were calculated for each city and province [35]. The population in each city and province was obtained from the household registered population [36].

### 2.4. Case Clustering in Different Locations

To compare the frequency of case clustering between locations, the clustering cases were counted for each of the 15 specific types of infection location listed in the third column in Table 1. The number of cases connected in Figure 1 was determined for each type of location. Note that the cases that connect multiple clusters were counted for each type of location. As a reference, the total exposure time of all reported cases for each type of location were also quantified. On traditional trains, electric multiple units (EMUs; a series of high-speed trains) or aeroplanes, the case’s total exposure time was calculated by searching for and summing all travel durations. For each of the other 12 location types, the total exposure time was estimated by the product of the total number of visits to the location and the average duration of stay per visit. In the 12 types of location, the average duration of stay per visit was assumed to be 12, 1 and 1 h for home, bus/metro and taxi/private car, respectively [37]; and those for the other nine types of location were estimated by averaging the durations of stay per visit in the corresponding type of location mentioned in the case reports.

### 2.5. Contact Times between Cases in Different Location Categories

We compared the clustering frequency of cases of different distances, i.e., contacts between cases whose home locations were separated as follows: inter-province contacts (connections between cases from different provinces in Figure 1), inter-city contacts (connections between cases from different cities in the same province in Figure 1) and intra-city contacts (connections between cases from the same city in Figure 1). However, a case could be clustered with cases from the same and other cities simultaneously. For this reason, we cannot associate every case with merely a single contact event. Thus, we assumed a series of rules, set out below, to enumerate the contacts between cases as a parameter to quantify the clustering frequency of cases of different distances. We introduced a definition, named effective contact, as the minimum number of contacts required for one source case to infect all the others in a cluster. For a cluster of *n* people that includes one infection source, the number of effective contacts equals *n* −1. The contacts counted in this study refer only to effective contacts, because extra contacts do not contribute to infection.

We first set a case as the initial infection source of COVID-19 for each cluster following the principles below. For a case in a cluster,

I. If a case is imported from Hubei, it is considered to be the infection source of the first order.
II. If no cases are imported from Hubei but one is imported from outside the three provinces of Northeast China, it is considered to be the infection source of the second order.
III. If no cases are imported from outside the three provinces of northeast China, but a case is imported that has been in close contact with some cases confirmed in other provinces, it is considered to be the infection source of the third order.
IV. If there are multiple possible infection sources of the same order, or if there are no possible transmission sources, the first reported case (usually the case with the lowest serial number) among the cases of the highest order is considered to be the infection source.

In addition, we set the priority order of contact between cases. For each infected case in the clusters in Figure 1 (i.e., for all cases except the assumed source cases), we selected an upstream case as its most likely contacted case following the principles below:

I. The disease spreads from the source case to the other cases step by step.
II. The case upstream from an infected case is only selected from direct-connected cases (without an intermediate case).
III. For an infected case with connected cases from different cities or provinces, the infection is first found from the cases of the same city, then from different cities in the same province, and then from different provinces.
IV. For any two cases with more than one type of contact, we assume that the infection occurs in the category of contact with a smaller order in Table 1.

Thus, for each cluster in Figure 1, we assumed one case to be the initial source of infection and determined an upstream contacted case for each of the other cases. The number of contacts was then counted respectively for each category of location (in Table 1) based on the rules introduced above. The contacts were further sub-categorised into inter-province contacts, inter-city contacts and intra-city contacts. The number of contacts in each sub-category was also counted. Note that the assumed contact network in each cluster can be considered as a possible transmission chain for each case cluster. However, the number of contacts, as our target parameter, is related only to the set priority order of effective contact, but independent of the choice of source cases and the consequent possible transmission chains. The assumption of a source case is made only for the convenience of counting the number of contacts.

## 3. Results

### 3.1. Regional Epidemic Situations

In general, Figure 1 shows that the epidemic situation varies by province and by city. The total number of cases, the number of clusters and the average scale of the clusters in Heilongjiang are generally greater than those in the other two provinces. In all three provinces, most cases had recorded contacts with other cases and were therefore grouped into case clusters. Inter-city and inter-province contact events occurred frequently. Five hundred and twenty-six of the 712 cases were clustered due to family gatherings at home. Most clusters have a simple structure with a single location of occurrence, but there are also some clusters in which multiple clustering events are connected to each other via several case nodes to form a larger cluster in which the exact infection sources are difficult to identify.

**Figure 2:**
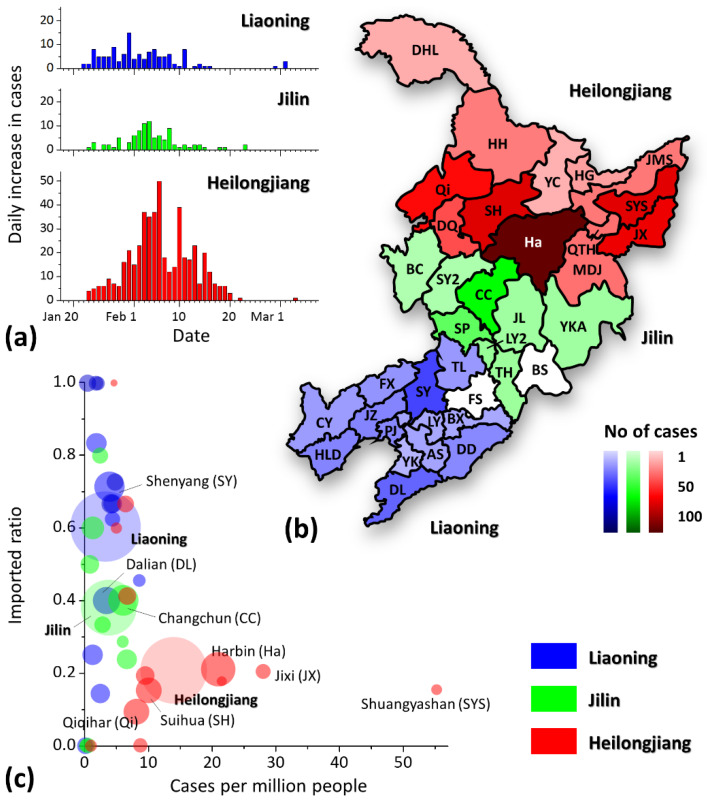
Epidemic situation in the three provinces (blue, Liaoning and its cities; green, Jilin and its cities; red, Heilongjiang and its cities). (**a**) Daily increase in the cases number from 18 January 2020 to 8 March 2020; (**b**) case map for the three provinces, in which the relative darkness of the cities represents the case number; and (**c**) epidemic situation of the three provinces and the included cities, in which the area of each city and province is proportional to its population.

The regional epidemic situations for all cities are shown in Figure 2. Figure 2a depicts the daily increase in cases in each province. The first case was reported on 22 January 2020 in each of the three provinces. The peak of the daily increase in cases occurred later in Heilongjiang than in the other two provinces, but the peak was significantly higher than in the other two provinces. In terms of the absolute number of cases, Harbin became the worst affected city, with the darkest red area in Figure 2b, and its cases comprised 38.5% of all cases in Heilongjiang and 26.7% in the three provinces. Figure 2c shows obvious differences in epidemic severity among cities and provinces. Cities on the horizontal axis (*y* = 0) received no imported cases and thus had a light epidemic burden, and cities on the line of *y* = 1 had no locally infected cases and thus showed good performance in disease prevention and control. The epidemic situation in some cities in Heilongjiang (red areas), such as Shuangyashan, Jixi and Harbin, is significantly more serious than in the other regions. The situations of Liaoning and Jilin were relatively better. Furthermore, a higher imported ratio was found in Liaoning than in Jilin (imported cases/total cases: Liaoning, 76/126; Jilin, 35/92; Heilongjiang, 102/494), which indicates a lower ratio of local community infection in Liaoning and thus a better effect of COVID-19 prevention and control.

### 3.2. Case Clustering in Various Locations

**Figure 3:**
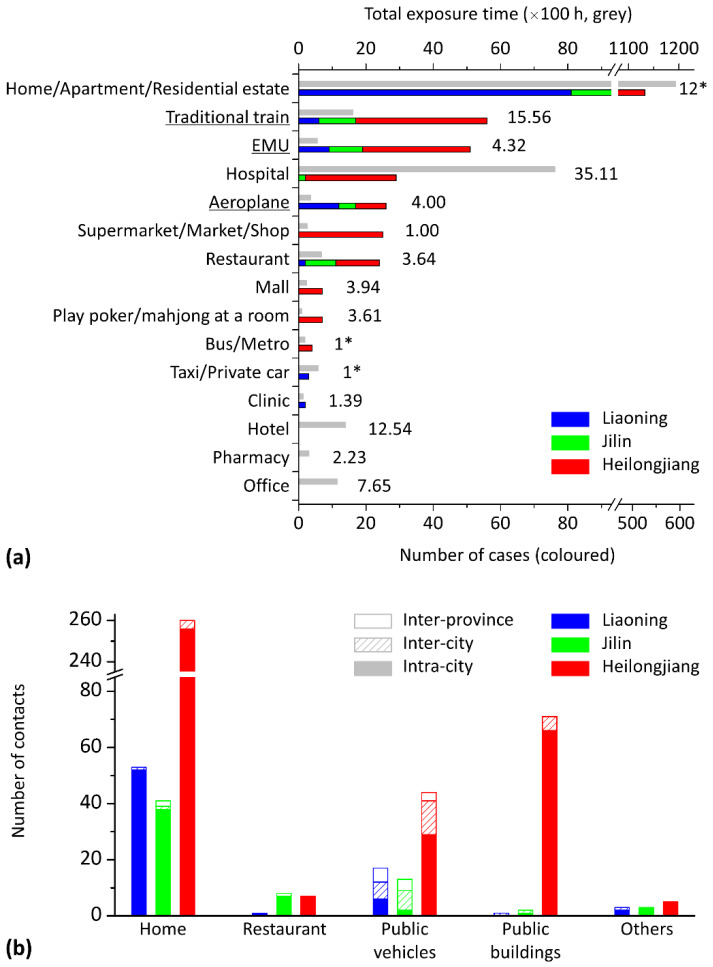
Statistical results of case clustering in various types of locations (blue, Liaoning; green, Jilin; red, Heilongjiang). (**a**) Number of cases clustered in the 15 types of location listed in Table 1. Each bar is a stack of cases from all three provinces. As a reference, the cases’ total exposure time (×100 h, grey bars) and unit exposure time (hours) are listed for each type of location. Three important types of public vehicle are underlined. The unit exposure times estimated from experience are marked with an asterisk. (**b**) Number of contacts between cases in five types of location. The contact events in each category were subcategorised into the provinces in which the contact occurred, and each bar is a stack of inter-province, inter-city and intra-city contacts.

The risk of infection in various locations is compared by counting the number of clustered cases in each type of location (in Figure 3a) and the contacts between cases of different distances (in Figure 3b). As shown in Figure 3a, 69.2% of total cases were clustered in a home, apartment or residential estate. Considering the lack of detailed information in the case reports, it is believed that some cases in the same apartment or residential estate could be from the same family. Some clusters also occurred in restaurants (3.2%) and public buildings (9.2%), such as hospitals, shops and malls. However, a high frequency of infection in a type of location could be due to long exposure times in such locations, rather than the infection being particularly efficient. In Figure 3a, the total exposure time of the 712 cases in each type of location is also displayed as a reference to compare with the number of cases clustered in the location. Apart from the location of home/apartment/residential estate, the cases also exposed themselves for a long time in hospitals, hotels, offices and the coaches of traditional trains.

As shown in Figure 3b, the number of contacts between cases of different distances (i.e., inter-province, inter-city and intra-city) varied by location category. In general, 89.8% of the contacts occurred between cases from the same city. The inter-city and inter-province contacts occurred mostly on public vehicles (68.5%). However, a few inter-province and inter-city contacts still occurred at home or in public buildings, as cases received friends or family from other cities or provinces. In each category of location in Figure 3b, the number of contacts also varies by province. The number of contacts between cases in public buildings in Heilongjiang was significantly higher than those in the other two provinces (1, 2 and 77 events in Liaoning, Jilin and Heilongjiang, respectively).

### 3.3. Case Contact on Public Transport

**Figure 4:**
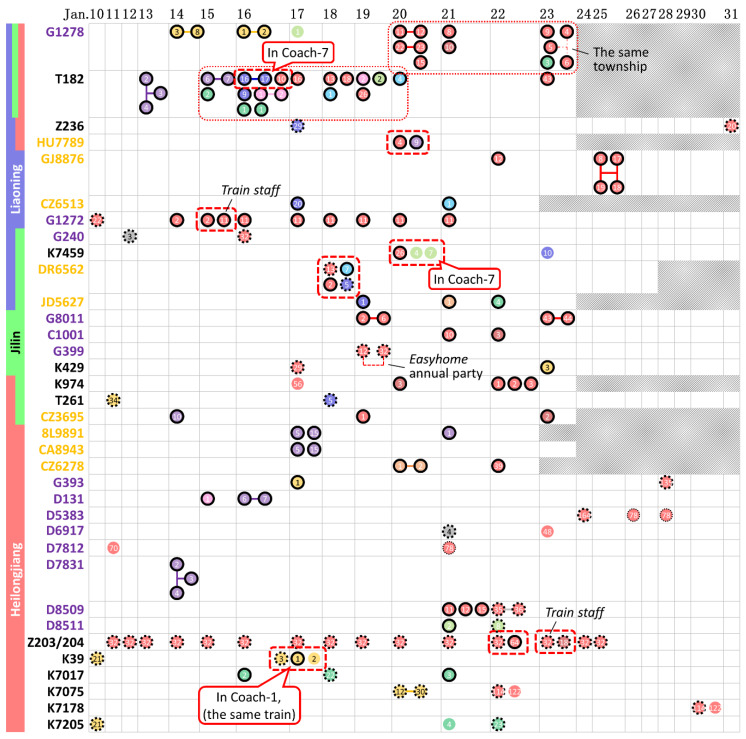
Diagram of cases clustering on trains (black, traditional trains; purple, EMUs) and aeroplanes (orange), in reference to the train and flight numbers and the travel date. Each ribbon with one or multiple colours shows the provinces of the cases source. The case cluster corresponding to each train or aeroplane can be found in Figure 1. The characteristics and the numbering of the icons are also consistent with those in Figure 1. The cases on the same coach/flight are enclosed by a dashed line. Grey shadows represent trains/flights that should have departed from Wuhan that were cancelled on that day (or the previous day for overnight trains).

Figure 4 lists all 35 public vehicles, including 12 traditional trains, 14 EMUs and 9 flights, that were taken by cases from different families. The trains G1278 and T182, both of which departed from Wuhan, suffered from the most severe epidemic situation and transported a number of cases to all three provinces. On the 35 public vehicles, contact occurred frequently between cases of each category of distance (inter-province, inter-city and intra-city contacts). However, only eight groups of cases were observed in the same coach of a train or on the same flight, as enclosed by dashed lines in Figure 4, and three groups of cases involved train staff. Overall, most cases had a history of residence in Hubei but travelled in different coaches or on different dates.

## 4. Discussion

During the period up to 8 March, the daily increase in cases in all three provinces reached its lowest point, and no new cases were confirmed from 5 to 9 March. The daily increase in cases rose again after 9 March, due to imported cases from overseas. We mainly analysed the effect of intervention in various regions and focused on the frequency of case clusters in various locations.

The epidemic situation significantly varied by city and province. The case density and the imported ratio were calculated for comparing the regional epidemic situations, as shown in Figure 2c, in which the cities and provinces to the upper left of the graph have lower case densities and local infection rates (i.e., a high imported ratio among the cases), which means that these cities are considered least affected; in contrast, the cities to the lower right of the graph encountered a heavier epidemic burden due to the higher case density and local infection rate. We mark all areas with a population of more than 5 million in Figure 2c, including six cities (Shenyang, Dalian, Changchun, Harbin, Qiqihar and Suihua) and the three provinces. Most of these cities’ positions in Figure 2c are very close to their provinces (except for Dalian, in which some cases did not have specified itineraries and were thus not counted as imported cases), which means that the epidemic situation in each province was highly related to that in these metropolises. We believe that this relationship was due not only to the cities’ large percentage of their provinces’ population, but also to their status as the transport hubs for the surrounding areas.

The differences in the epidemic situation between cities and provinces may be due to differences in government actions and in public awareness of epidemic prevention and control. All three provinces initiated a Level I Emergency Response to the public health emergency on 25 January 2020. However, several clustering events occurred in public buildings in Heilongjiang after that date (Figure 1). Considering the similar natural environment, culture and economic development level in the three adjacent provinces, it may be hypothesized that local government actions and the efficiency in intervention played a major role in the epidemic among regions [38] and that the early implementation of social distancing, testing and diagnostics can have a significant impact on epidemics [3,9,39]. In addition, as shown in Figure 1, significantly more large-family clusters (involving relatives from different estates) were found in Heilongjiang, which means that people continued visiting relatives during this severe epidemic, indicating an absence of public awareness of epidemic prevention relative to the other two provinces.

Case clusters mostly occurred at home between families or relatives. In general, infection events on vehicles and in families were difficult to avoid at the beginning of the COVID-19 outbreak. The outbreak overlapped with the 2020 Lunar New Year holidays (24 January to 2 February), for which people travelled home to reunite with their families [40]. Some imported cases were quarantining themselves at home with their families before their diagnosis.

However, the high prevalence of cases in some cities in Figure 2c was mainly due to the occurrence of several local outbreaks in public buildings, as cases who had been newly infected in public buildings could then act as a source to infect their families. Infection in public buildings usually occurred between non-associated individuals or friends from different families. Once infection occurred, it could further lead to an outbreak in new families via the newly infected cases [41,42]. Some cities had a significantly large case density, such as Harbin, Jixi, Suihua, Shuangyashan and Changchun (Figure 2c). We have observed large scale of clusters in public buildings in all these cities. For instance, seven cases from Harbin and Suihua, in Heilongjiang, were staff or shoppers from Toulong Mall (a Heilongjiang cluster with red and blue icons in Figure 1); some were also members of family clusters, which indicates that the cases from Toulong Mall could be the second-generation infection sources of their family clustering infections; similar local outbreaks were also found in Qiqihar First Hospital (a Heilongjiang cluster with grey icons in Figure 1), the Easyhome company’s annual party (a Jilin cluster with red icons in Figure 1) and three fruit markets in Shuangyashan (a Heilongjiang cluster with green icons in Figure 1). Thus, public buildings showed a great effect on the spread of COVID-19.

In some cases, the patients went to the same hotels or pharmacies or took the same buses or cars, but very few cases were confirmed from those categories of locations because the summarised case clustering in those locations all occurred between families or relatives, so we believe that their infection is more likely to have occurred at home. Clustering occurred far less often in clinics than in hospitals, possibly due to the shorter average unit exposure time (shown in Figure 3a), as clinics usually have no long-term inpatients. In addition, no cases were found to have been infected in offices, except for staff working in hospitals and clinics.

Some cases were clustered in public transport (17.5%). As shown in Figure 3b, contacts on public transport led to more connections between cases from different cities or provinces than contacts in public buildings or at home. This means that contact between people as they travel by (or queue for) public transport could result in infection, spreading the disease to a new area. Passengers on vehicles have a high risk of infection, but the risk still varies by the vehicle type. A higher infection rate was seen on traditional trains than on EMUs and aeroplanes. The case reports included 104 trips on traditional trains, and we found 56 clustering cases; the ratio of the number of clustering cases to the total number of reported trips was thus 53.8%. This ratio was 39.5% on EMUs and 28.6% on aeroplanes, possibly due to the long average unit exposure time on traditional trains (15.56 h; shown in Figure 3a) compared with EMUs (4.32 h) and aeroplanes (4.00 h). In addition, the direction of seats (and beds) may also influence the infection risk. Passengers in aeroplane cabins and most EMU coaches face forward, whilst the seats and beds on traditional trains face each other [43].

Although Figure 1 and Figure 3 show a significant number of clusters on public vehicles, the number of actual infection events on trains and aeroplanes may have been far lower than the number of contacts quantified in Figure 3b. Figure 1 shows that a significant proportion of cases on the clustering trains and aeroplanes had a residence history in Hubei (icons with black solid edge), the site of the initial outbreak. Figure 4 further summarises the trains and aeroplanes in which cases from different families clustered. Eleven of the 35 trains or flights had cases from different provinces. The two trains, G1278 and T182, departing from Wuhan included patients from all three provinces. Some cases on the two trains travelled on the same day (enclosed with thin-dot line in Figure 4), which means that the infection could have occurred in the train coaches or in the trains’ waiting rooms.

However, we found only eight groups of cases in Figure 4 (enclosed with dashed line), each as a cluster with cases from different families clustering in the same coach of a train or the cabin of the same flight. There was a high probability of close contact between the cases in each group. In contrast, most cases had a residence history in Hubei Province and travelled on different dates, which means that the cases that departed from Hubei may have been infected before they boarded the trains or aeroplanes. Thus, we still believe that public vehicles are risky locations where cases tend to cluster, but not all cases were infected during their trips. Public vehicles play two roles, as the locations in which infection occurs and as the agents that transport cases to a new area. Some cases were truly infected during the trips, whilst most cases were purely clustered by the trains or aeroplanes with no infection occurring during transportation to a new area [44].

In addition, 76 cases or case clusters in Figure 1 had no connection to any imported cases or contact with any known COVID-19 sources. In addition to the incomplete nature of the itinerary report, we believe that the absence of a possible infection source for a locally infected case (or case cluster) is due to the presence of asymptomatic cases [17,30]. For example, the case Fuxin-6 in Liaoning Province was reported to have had contact with a relative who took the train G1274, one of whose on-board staff became a COVID-19 confirmed case (Shenyang-11), but the relative of Fuxin-6 was not reported as a confirmed case. If we assume that each case (or case cluster) with no connection to any imported sources was infected via an asymptomatic case [14], then there should be about 76 asymptomatic cases, which yields an estimated ratio of asymptomatic cases to all cases of 76/(76 + 712) = 9.6%. This ratio is higher than the corresponding ratio of 5.0% in Beijing [45], which indicates that potential asymptomatic cases in the crowd might have not been detected and confirmed. Furthermore, some cases had family members who were not named as infection sources in the case reports but may have been asymptomatic carriers, despite being officially excluded as confirmed cases. Thus, we suspect that the ratio of the number of asymptomatic cases to symptomatic cases might be actually much higher than 9.6%, which is also evidenced by some recent reports [28,46,47].

## 5. Conclusions

This study analysed the epidemic situations in various areas in the three provinces of Northeast China and the effects of various types of infection location on identified COVID-19 clusters. In general, the epidemic situations varied significantly by city and province. Most infections occurred between family members and relatives at home, and their contacts usually involved dining activities. Case clustering in public buildings might play a hub role in regional epidemic, such as in Heilongjiang, because clustering events could cause infection between different families, which could cause further clusters of infection in more families. Some case clusters occurred on trains and aeroplanes, which transported the cases to new areas, and this situation also increased the risk of infection during the trip. The timely closure of public buildings and efforts to raise public awareness may help to significantly relieve regional epidemics in the future.

## Figures and Tables

**Table 1:** Categories of infection location by the contact activities involved.

No.	Categories	Infection Locations	Activities Involved
1	Home	Estate (own home/relative’s home)	Dining and long-term or repeated household close contacts
2	Restaurant	Restaurant	Dining
3	Public vehicles	Traditional train; EMUs; aeroplane; taxi/private car; bus/metro.	Long-term close contacts
4	Public buildings	Residence (apartment/residential estate/friend’s home); supermarket/market/shop; hotel; hospital; clinic; pharmacy; mall; office; poker/mahjong room	Close contacts
5	Others	Infection locations cannot be determined	Unknown

## Supplementary Materials

The following are available online at https://www.mdpi.com/1660-4601/17/11/3955/s1, Table S1: Statistic result of 712 cases.
